# A multilevel analysis of the importance of oral health instructions for preventing tooth loss: The 8020 Promotion Foundation Study of Japanese Dental Patients

**DOI:** 10.1186/s12903-020-01319-9

**Published:** 2020-11-18

**Authors:** Mizuki Saito, Yoshihiro Shimazaki, Kakuhiro Fukai, Michiko Furuta, Jun Aida, Yuichi Ando, Hideo Miyazaki, Masaki Kambara

**Affiliations:** 1 Department of Preventive Dentistry and Dental Public Health, School of Dentistry, Aichi Gakuin University, 1-100 Kusumoto-cho, Chikusa-ku, Nagoya, 464-8650 Japan; 2 8020 Promotion Foundation, Tokyo, Japan; 3 Fukai Institute of Health Science, Misato, Japan; 4 Section of Preventive and Public Health Dentistry, Kyushu University Faculty of Dental Science, Fukuoka, Japan; 5 Department of International and Community Oral Health, Tohoku University Graduate School of Dentistry, Sendai, Japan; 6 National Institute of Public Health, Wako, Japan; 7 Department of Oral Health Science, Niigata University Graduate School of Medical and Dental Science, Niigata, Japan; 8 Osaka Dental University, Hirakata, Japan

**Keywords:** Multilevel analysis, Tooth loss, Oral health instruction, Dental hygienist, Dental patients

## Abstract

**Background:**

Many studies have reported risk factors for tooth loss. Oral health instruction is considered effective at improving oral health behavior and oral health. However, few studies have examined the relationship of dental clinic factors, such as the number of dental hygienists and implementation of oral health instructions, with tooth loss. Here, we conducted a multilevel analysis to clarify the dental clinic risk factors associated with tooth loss.

**Methods:**

Baseline surveys were conducted at 1216 dental clinics in 46 prefectures in Japan, and 12,399 dental patients aged 20 years and over underwent oral examinations and completed a questionnaire. The dental clinics also completed a questionnaire at baseline. A 3-year follow-up survey included 2488 patients in 585 dental clinics. Multilevel multivariate logistic regression analysis was used to examine the risk of tooth loss at the patient and clinic levels.

**Results:**

Of the patient variables, older age, higher mean probing pocket depth, current or past smoking, and bleeding during tooth brushing were associated with higher risks of tooth loss. Individuals with many teeth who visited dental clinics for maintenance were at significantly lower risk of tooth loss. Of the clinic variables, patients attending dental clinics with four or more dental hygienists had a significantly lower risk of tooth loss (OR 0.68, 95% CI 0.50–0.99). Patients attending dental clinics that provide oral health instructions for 20 min or more had a significantly lower risk of tooth loss (OR 0.69, 95% CI 0.50–0.96).

**Conclusions:**

In addition to individual risk factors for tooth loss, dental clinic factors such as length of oral health instruction and number of dental hygienists are associated with tooth loss. In dental clinics, ensuring sufficient time for dental hygienists to provide oral health instructions can help prevent tooth loss in dental patients.

## Background

In Japan, adults are losing fewer teeth, although older people still lose many teeth [1]. Tooth loss affects masticatory and swallowing function, conversation, and appearance. Especially in older people, tooth loss is an important problem associated with a poor quality of life because the loss of many teeth leads to malnutrition, resulting in reduced activities of daily living and dementia [2]. Preventing the deterioration of chewing ability and swallowing function by retaining many teeth may help to extend the healthy lifespan [3, 4]. A study of people older than 75 years showed that those with fewer teeth had higher medical costs associated with stroke [5]. Preventing tooth loss may help to curb medical expenses [6].

Many studies have reported the risks of tooth loss [7–9]. Since individual- and tooth-level factors are involved in tooth loss, some studies have conducted multilevel analyses to identify the risk of tooth loss [10, 11]. Tooth loss is associated with the type of dental visit and the risk of tooth loss was lower in those who had regular dental checkups compared with those who received treatment only [12, 13]. The number of people visiting dental clinics for dental checkups is increasing [14]. Therefore, it is important to clarify the dental-clinic factors related to dental visits. For example, oral health instructions improve oral health behavior and oral health [15, 16]. However, few studies have examined the relationships of dental-clinic factors, such as the number of dental hygienists and the provision of oral health instructions, with tooth loss.

In this study, we conducted a multilevel analysis to clarify the dental-clinic factors related to tooth loss. We examined both patient and clinic factors, including the number of dental hygienists and the time spent on oral health instructions, affecting tooth loss among dental patients aged 20 years or older.

## Methods

### Population

**Fig. 1:**
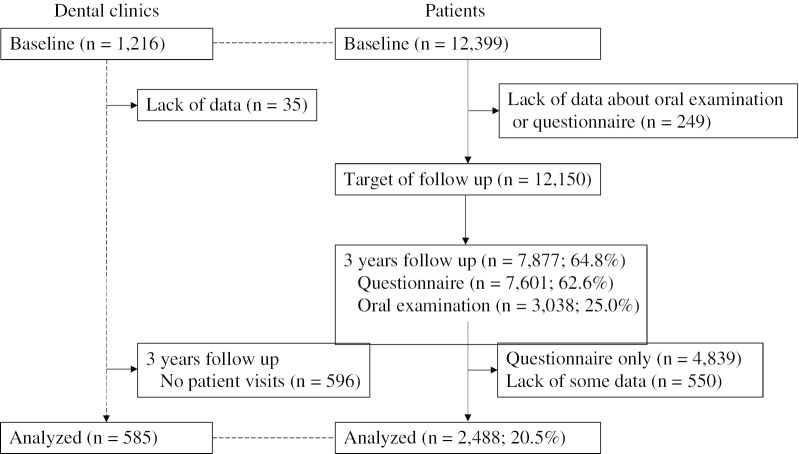
Flowchart of dental clinics and patients

This study used data from the 8020 Promotion Foundation Study of Japanese Dental Patients. The survey was conducted in dental clinics and adult dental patients across the country were enrolled to determine the health-promoting effects of dental care. Baseline surveys were conducted at 1216 dental clinics in 46 prefectures in Japan. During any one week in October 2014, 12,399 people aged 20 and over who visited dental clinics for a first visit or revisit more 2 months after their last visit underwent an oral examination and completed a questionnaire. Of these, we enrolled 12,150 people with complete oral examination and questionnaire data. Each year, we mailed all of them the same questionnaire and dental checkup form as at baseline and asked them to return it after they had completed it. Data on the oral examinations were collected if they visited a dental clinic during the follow-up period, but we did not encourage them to visit a dental clinic for the purpose our study. Of the baseline participants, those participating in follow-up survey were not necessarily matched in each year, and follow-up rates varied by year. For this study, we analyzed the questionnaire and oral examination at baseline and the 3-year follow-up oral examination from October 31, 2017 to March 31, 2018. Of the 12,150 patients, 7877 (64.8%) participated in the 3-year follow-up, 7601 (62.6%) responded to the questionnaire, and 3038 (25.0%) underwent follow-up oral examinations at dental clinics (Fig. 1).

The Ethics Committee of the Japanese Association for Dental Science, approved the study design, data-collection methods, and procedure for obtaining informed consent (Approval No. 0002); informed consent was obtained from all participants.

### Oral health examination

The oral examination was performed by a dentist at dental clinics belonging to the Japan Dental Association, and teeth and periodontal tissues were evaluated. Tooth condition was recorded as sound, decayed, filled, or missing; the number of teeth was calculated as the sum of sound, decayed, and filled teeth, excluding third molars. The periodontal tissue examination evaluated probing pocket depth (PPD) and bleeding during probing (BOP) at six points (buccal mesial, buccal central, buccal distal, lingual mesial, lingual central, and lingual distal) on each tooth.

### Questionnaire

The questionnaire items for patients included smoking habit (never, past, current), history of diabetes (no, yes), tooth brushing frequency (≥ 3, 2, or ≤ 1 times per day), use of interdental cleaning tools such as dental floss or interdental brush (no, yes), bleeding from gums when brushing teeth (no, yes), eating between meals (no, sometimes, everyday), reason for dental visit (treatment, both treatment and maintenance, maintenance), education level (≥ 13 or ≤ 12 years), subjective economic status (lower, middle, upper), and working status (no, yes) (see Additional file 1).

### Dental clinic survey

The dental clinic questionnaire at baseline asked about the gender and age of the clinic director (30 s, 40 s, 50 s, 60 s, 70 s or older), number of dental hygienists, and amount of time spent on oral health instructions (0, 1–4, 5–9, 10–19, 20–29, ≥ 30 min) (see Additional file 1). We did not ask for details of the oral health instructions.

### Analysis

We analyzed 2,488 patients seen in 585 dental clinics, excluding 4,839 patients with only questionnaire data at follow-up and 550 patients who lacked some data. Those who had a decrease of one or more teeth between baseline and follow-up were counted as those who had lost teeth. The age of each dental clinic director was divided into three categories (30 s or 40 s, 50 s, and 60 s or older). The number of dental hygienists was classified into three categories (0, 1–3, and ≥ 4). The time spent on oral health instructions was divided into four categories (0, 1–9, 10–19, and ≥ 20 min). The mean PPD of teeth examined and proportion of BOP-positive teeth were used as indexes of periodontal condition. To analyze the differences in the mean or proportion of each variable, analysis of variance was used for continuous data and the chi-square test for categorical data. To analyze the stratified data, a multilevel logistic regression analysis based on the generalized estimation equation specifying the distribution and link function was used to examine the risk of tooth loss at the patient and clinic levels by calculating the odds ratio (OR) and 95% confidence intervals (CIs). The presence or absence of tooth loss was used as the dependent variable and the independent variables included patient (age, gender, number of teeth, number of decayed teeth, mean PPD, BOP, smoking, history of diabetes, tooth brushing frequency, use of interdental cleaning tools, bleeding from gums, eating between meals, reason for dental visit, education level, subjective economic status, and working status) and clinic (age and gender of dental clinic director, number of dental hygienists, and amount of time spent on oral health instructions) variables. All analyses were conducted using SPSS ver. 24.0 (IBM, Armonk, NY, USA). *P* < 0.05 was considered indicative of statistical significance.

## Results

In the targeted clinics, 95% of the directors were male, and most directors were in their 50 s. Approximately two-thirds of the dental clinics had one to three dental hygienists, and dental clinics providing 1–9 or 10–19 min of oral health instructions each accounted for a third.

Table 1 shows the association between the presence or absence of tooth loss and each variable. Of the participants, 691 (27.8%) lost one or more teeth during the 3-year period. Of the patient variables, age, numbers of teeth and decayed teeth, mean PPD, BOP, gender, smoking, history of diabetes mellitus, tooth brushing frequency, bleeding during tooth brushing, eating between meals, reason for dental visit, education level, and working status were significantly associated with tooth loss. Of the clinic variables, director’s age, number of dental hygienists, and time spent on oral health instructions were significantly associated with tooth loss. The percentage of patients with tooth loss was 30.4% for patients in dental clinics without dental hygienists compared to 21.3% for patients in dental clinics with four or more dental hygienists. The percentage of tooth loss among patients in dental clinics that did not provide oral health instruction was 31.5%, whereas the percentage among patients in dental clinics with ≥ 20 min of instruction was 23.0%.

**Table 1:** Baseline characteristics of patients and their dental clinics according to tooth loss

	Tooth loss		*p* value
	Absence (n = 1797)	Presence (n = 691)	
Patient-level variables	Mean (SD)		
Age (years)	57.0 (14.7)	65.5 (11.2)	< 0.001
Number of teeth	24.9 (4.5)	21.2 (6.0)	< 0.001
Number of decayed teeth	0.4 (1.2)	0.6 (1.4)	0.009
Mean PPD (mm)	2.8 (0.8)	3.2 (1.0)	< 0.001
BOP (%)	23.3 (25.5)	30.4 (28.5)	< 0.001
n (%)			
Gender			
Male	549 (67.3)	267 (32.7)	< 0.001
Female	1248 (74.6)	424 (25.4)	
Smoking			
Never	1307 (75.2)	430 (24.8)	< 0.001
Past	362 (65.5)	191 (34.5)	
Current	128 (64.6)	70 (35.4)	
Diabetes mellitus			
No	1702 (72.9)	632 (27.1)	0.002
Yes	95 (61.7)	59 (38.3)	
Tooth brushing frequency (times per day)			
≥ 3	685 (76.8)	207 (23.2)	< 0.001
2	917 (70.8)	379 (29.2)	
≤ 1	195 (65.0)	105 (35.0)	
Use of secondary oral hygiene products^a^			
Yes	1356 (73.0)	502 (27.0)	0.082
No	441 (70.0)	189 (30.0)	
Bleeding during tooth brushing			
No	1,192 (75.6)	385 (24.4)	< 0.001
Yes	605 (66.4)	306 (33.6)	
Eating between meals			
No	205(63.5)	118 (36.5)	< 0.001
Sometimes	986 (72.5)	374 (27.5)	
Everyday	606 (75.3)	199 (24.7)	
Reason for dental visit			
Treatment	527 (63.0)	309 (37.0)	< 0.001
Both treatment and maintenance	313 (68.6)	143 (31.4)	
Maintenance	957 (80.0)	239 (20.0)	
Education level (years)			
≥ 13	933 (77.2)	276 (22.8)	< 0.001
≤ 12	868 (67.6)	415 (32.4)	
Economic status			
Low	307 (72.1)	119 (27.9)	0.728
Middle	1,241 (72.6)	468 (27.4)	
High	249 (70.5)	104 (29.5)	
Working status			
Yes	1,079 (77.9)	306 (22.1)	< 0.001
No	718 (65.1)	385 (34.9)	
Clinic-level variables^b^			
Age of dental clinic director			
30s or 40s	600 (76.5)	184 (23.5)	0.005
50s	907 (70.5)	380 (29.5)	
60s or over	290 (69.5)	127 (30.5)	
Gender of dental clinic director			
Male	1,680 (72.0)	653 (28.0)	0.201
Female	117 (75.5)	38 (24.5)	
Number of dental hygienists			
0	295 (69.6)	129 (30.4)	0.005
1–3	1,199 (71.4)	478 (28.6)	
≥ 4	311 (78.7)	84 (21.3)	
Time for oral health instruction (min)			
0	243 (68.5)	112 (31.5)	0.003
1–9	570 (69.0)	256 (31.0)	
10–19	613 (74.3)	212 (25.7)	
≥ 20	371 (77.0)	111 (23.0)	

*SD* standard deviation, *PPD* probing pocket depth, *BOP* breeding on probing

^a^Dental floss or interdental brush

^b^These are the clinic information for each patient based on linking clinic data to patient data

Table 2 shows the results of the multilevel analysis. Of the patient variables, older age, higher mean PPD, current or past smoking, and bleeding during tooth brushing had higher risks of tooth loss. Individuals with many teeth and who visited a dental clinic for maintenance were at significantly lower risk of tooth loss. Of the clinic variables, the patients attending dental clinics with four or more dental hygienists had a significantly lower risk of tooth loss (adjusted OR 0.68, 95% CI 0.50–0.99) compared to those attending dental clinics without dental hygienists. Moreover, the patients attending dental clinics that provided oral health instructions for 20 min or more had a significantly lower risk of tooth loss (adjusted OR 0.69, 95% CI 0.50–0.96) compared to those attending dental clinics that did not provide oral health instructions.

**Table 2:** Multilevel multivariate generalized estimation equation (logistic regression analysis) for relationship of patient- and clinic-level variables with tooth loss

	Dependent variable: Tooth loss (absence = 0, presence = 1)			
	Crude OR (95%CI)	*p* value	Adjusted OR (95%CI)	*p* value
Patient-level variables				
Age (years)	1.05 (1.04–1.06)	< 0.001	1.03 (1.02–1.04)	< 0.001
Number of teeth	0.88 (0.87–0.90)	< 0.001	0.93 (0.91–0.95)	< 0.001
Number of decayed teeth	1.09 (1.02–1.16)	0.011	1.04 (0.97–1.12)	0.253
Mean PPD (mm)	1.76 (1.58–1.96)	< 0.001	1.46 (1.26–1.68)	< 0.001
BOP (%)	1.01 (1.01–1.01)	< 0.001	1.00 (1.00–1.00)	0.917
Gender				
Male	1		1	
Female	0.70 (0.58–0.84)	< 0.001	1.21 (0.92–1.60)	0.177
Smoking				
Never	1		1	
Past	1.60 (1.31–1.97)	< 0.001	1.55 (1.19–2.01)	0.001
Current	1.66 (1.22–2.27)	0.001	1.67 (1.13–2.47)	0.011
Diabetes mellitus				
No	1		1	
Yes	1.67 (1.19–2.34)	0.003	1.07 (0.75–1.53)	0.727
Tooth brushing frequency (times per day)				
≥ 3	1		1	
2	1.37 (1.12–1.66)	0.002	1.09 (0.87–1.35)	0.456
≤ 1	1.78 (1.34–2.37)	< 0.001	1.11 (0.81–1.53)	0.525
Use of secondary oral hygiene products^a^				
Yes	1		1	
No	1.16 (0.95–1.41)	0.149	0.83 (0.65–1.06)	0.134
Bleeding during tooth brushing				
No	1		1	
Yes	1.57 (1.31–1.87)	< 0.001	1.51 (1.23–1.85)	< 0.001
Eating between meals				
No	1		1	
Sometimes	0.66 (0.51–0.85)	0.001	0.77 (0.58–1.02)	0.067
Everyday	0.57 (0.43–0.75)	< 0.001	0.75 (0.53–1.05)	0.094
Reason for dental visit				
Treatment	1		1	
Both treatment and maintenance	0.78 (0.61–0.99)	0.044	0.74 (0.56–0.98)	0.038
Maintenance	0.43 (0.35–0.52)	< 0.001	0.57 (0.45–0.71)	< 0.001
Education level (years)				
≥ 13	1		1	
≤ 12	0.62 (0.52–0.74)	< 0.001	0.95 (0.77–1.18)	0.643
Economic status				
Low	1		1	
Middle	0.97 (0.77–1.23)	0.820	0.98 (0.75–1.29)	0.881
High	1.08 (0.79–1.47)	0.639	1.11 (0.76–1.63)	0.582
Working status				
Yes	1		1	
No	1.89 (1.58–2.26)	< 0.001	1.18 (0.93–1.50)	0.180
Clinic-level variables				
Age of dental clinic director				
30s or 40s	1		1	
50s	1.37 (1.11–1.68)	0.003	1.28 (1.00–1.64)	0.052
60s or over	1.43 (1.09–1.86)	0.009	1.28 (0.93–1.75)	0.126
Gender of dental clinic director				
Male	1		1	
Female	0.84 (0.57–1.22)	0.350	0.73 (0.48–1.14)	0.164
Number of dental hygienists				
0	1		1	
1–3	0.92 (0.73–1.16)	0.470	0.99 (0.77–1.27)	0.922
≥ 4	0.62 (0.45–0.85)	0.003	0.68 (0.50–0.99)	0.047
Time for oral health instruction (min)				
0	1		1	
1–9	0.97 (0.75–1.27)	0.850	1.03 (0.76–1.40)	0.861
10–19	0.75 (0.57–0.99)	0.039	0.79 (0.57–1.08)	0.140
≥ 20	0.65 (0.48–0.88)	0.006	0.69 (0.50–0.96)	0.028

OR, odds ratio; CI, confidence interval; PPD, probing pocket depth; BOP, breeding on probing

^a^Dental floss or interdental brush

Table 3 shows the association between the time spent on oral health instructions and each variable. Patients attending the dental clinics that spent a long time on oral health instruction tended to use interdental cleaning tools and visit dental clinics for maintenance. The more dental hygienists that worked in a dental clinic, the longer the time spent on oral health instructions.

**Table 3:** Association of characteristics of patients and their dental clinics with time for oral health instruction

	Amount of time for oral health instruction (min)^a^				*p* value
	0	1–9	10–19	≥ 20	
Patient-level variables ^b^					
Smoking					
Never	245 (14.1)	591 (34.0)	567 (32.6)	334 (19.2)	0.536
Past	74 (13.4)	179 (32.4)	189 (34.2)	111 (20.1)	
Current	36 (18.2)	56 (28.3)	69 (34.8)	37 (18.7)	
Tooth brushing frequency (times per day)					
≥ 3	121 (13.6)	296 (33.2)	296 (33.2)	179 (20.1)	0.091
2	175 (13.5)	432 (33.3)	430 (33.2)	259 (20.0)	
≤ 1	59 (19.7)	98 (32.7)	99 (33.0)	44 (14.7)	
Eating between meals					
No	50 (15.5)	109 (33.7)	101 (31.3)	63 (19.5)	0.960
Sometimes	196 (14.4)	453 (33.3)	447 (32.9)	264 (19.4)	
Everyday	109 (13.5)	264 (32.8)	277 (34.4)	155 (19.3)	
Use of secondary oral hygiene products^c^					
Yes	215 (11.6)	634 (34.1)	630 (33.9)	379 (20.4)	< 0.001
No	140 (22.2)	192 (30.5)	195 (31.0)	103 (16.3)	
Bleeding of tooth brushing					
No	220 (14.0)	528 (33.5)	531 (33.7)	298 (18.9)	0.740
Yes	135 (14.8)	298 (32.7)	294 (32.3)	184 (20.2)	
Reason for dental visit					
Treatment	174 (20.8)	268 (32.1)	257 (30.7)	137 (16.4)	< 0.001
Both treatment and maintenance	60 (13.2)	144 (31.6)	165 (36.2)	87 (19.1)	
Maintenance	121 (10.1)	414 (34.6)	403 (33.7)	258 (21.6)	
Clinic-level variables^d^					
Age of dental clinic director					
30s or 40s	34 (15.7)	66 (30.4)	80 (36.9)	37 (17.1)	0.930
50s	46 (16.7)	92 (33.3)	87 (31.5)	51 (18.5)	
60s or over	15 (16.3)	32 (34.8)	29 (31.5)	16 (17.4)	
Gender of dental clinic director					
Male	93 (16.7)	180 (32.3)	189 (33.9)	96 (17.2)	0.234
Female	2 (7.4)	10 (37.0)	7 (25.9)	8 (29.6)	
Number of dental hygienists					
0	31 (26.3)	46 (39.0)	25 (21.2)	16 (13.6)	0.001
1–3	57 (14.8)	119 (30.9)	135 (35.1)	74 (19.2)	
≥ 4	7 (8.5)	25 (30.5)	36 (43.9)	14 (17.1)	

^a^This is based on a clinic-level variable

^b^Number of patients (n = 2488)

^c^Dental floss or interdental brush

^d^Number of dental clinics (n = 585)

## Discussion

This study examined the risk of tooth loss among dental clinic patients using a multilevel analysis including patient and clinic factors. Our results suggested that independent of patient factors, the number of dental hygienists and the time spent on oral health instructions were clinic factors significantly associated with tooth loss.

Of the factors associated with tooth loss, many patient factors were consistent with previous reports [8, 11, 17]. The uniqueness of this study is that it performed a multilevel analysis using patient and clinic variables for tooth loss. Of the clinic variables, the time spent on oral health instructions in the dental clinic was significantly associated with tooth loss among dental patients. Patients attending dental clinics that provided oral health instruction for at least 20 min had a significantly lower risk of tooth loss than those attending dental clinics that did not provide oral health instruction. One workplace study showed that workers receiving dental health instruction showed improved dental health behaviors, such as tooth brushing habits and the use of fluoridated toothpaste [15]. An intervention study of patients with periodontal disease reported that oral health instruction improved oral cleaning habits, oral cleaning status, and gingival status [16]. Setting self-care goals and providing self-monitoring and planning instruction is effective at improving oral cleaning habits in patients with periodontal disease [18, 19]. In this study, since we examined only the time spent on oral health instructions in each dental clinic, the content of the instructions was not known. Nevertheless, providing oral health instructions will improve the oral health habits and oral health of dental patients, which will help reduce tooth loss. It is important to provide sufficient time for oral health instructions for patients undergoing dental examinations.

In this study, patients who visited dental clinics that devoted considerable time to oral health instruction tended to use interdental cleaning tools and visited the dental clinic for maintenance. Using an interdental cleaning tool is effective for removing interdental plaque and for preventing dental caries and periodontal disease [20]. Since the main causes of tooth loss in adults are periodontal disease and dental caries [21], preventing their occurrence and progression will prevent tooth loss. In a study of the relationship between dental visits and caries, patients who made regular dental visits had significantly fewer caries and decayed and missing teeth [22]. It is believed that regular dental examinations lead to the prevention, early detection, and suppression of deterioration of dental caries. Therefore, recommending the use of interdental cleaning tools and explaining the importance of regular dental checkups while providing oral health instructions will help prevent tooth loss.

Of the clinic variables, the presence of four or more dental hygienists appeared to reduce the risk of tooth loss among the dental patients. In addition, dental clinics without a dental hygienist often did not provide oral health instructions. Dental hygienists can contribute to prevention of tooth loss by providing oral health instructions, such as tooth brushing instructions. Dental hygienists provide preventive measures such as fluoride application and tartar removal. If a clinic has many dental hygienists, it should be possible to provide sufficient time for oral health instructions. The presence of many dental hygienists also makes it easier to perform preventive dental measures and tooth loss can be suppressed by preventing dental diseases. The employment rate of dental hygienists in Japan is approximately 60%, where there is a shortage of dental hygienists because many have left their jobs due to marriage or childbirth [23]. If it is possible to increase the number of working dental hygienists by implementing training systems for returning to work and creating a comfortable working environment with flexible working hours, it would contribute to improving the oral health of dental patients.

As patient factors, those who smoked at baseline had a significantly higher risk of tooth loss than nonsmokers. Smoking is an important risk factor for periodontal disease [24]. Smokers respond poorly to basic periodontal treatment and surgery [25]. Moreover, smoking is associated with tooth loss [26], and the aggravation of periodontal disease by smoking is thought to increase the risk of tooth loss. It may be possible to reduce the risk of tooth loss among smokers by actively providing smoking cessation assistance in addition to oral health instruction at dental clinics.

Bleeding during tooth brushing may be due to periodontal inflammation or inappropriate oral hygiene habits. Those who bled while tooth brushing had a significantly higher risk of tooth loss. Paying attention to the results of oral examinations and the patient’s symptoms assists when providing oral health instructions.

This study has some limitations and advantages. The initial participants were those who had visited dental clinics during a limited period and the results might differ from the general population. The follow-up rate of this study was 20.5%, which did not reflect the overall condition of the baseline participants. The follow-up period was 3 years, but we could not assess the dental treatment or dental consultation status of the participants during the period. In addition, the causes of lost teeth were unknown. In this study, we used the presence or absence of tooth loss as the dependent variable. Therefore, those who lost one tooth and those who lost many teeth were treated equally in the model and the effect of the number of teeth lost could not be considered. There was also no information on the specific content of the oral health instructions and the time spent was a general answer for each dental clinic, so the specific time for each patient is unknown. Because we did not assess the participants’ oral hygiene status in the oral examination, we could not determine whether the oral health instructions led to improvements in their oral health status. There was no information on geographic factors, such as the distance between the patients’ residence and dental clinics, and it is possible that these factors may have influenced the patients’ visitation behavior. Although this study was conducted throughout Japan, the calibration among oral examiners was insufficient. The strength of this study is that study information was collected from many dental clinics in most prefectures in Japan and it had a large sample size. Despite its limitations, this study provides useful information about the risk of tooth loss in dental patients.

## Conclusion

In addition to patient factors affecting tooth loss, patients visiting dental clinics providing longer oral health instructions and with many dental hygienists have a lower risk of tooth loss. In dental clinics, ensuring sufficient time for oral health instructions by dental hygienists should help to prevent tooth loss in dental patients. Since tooth loss is compounded by several relevant factors and oral health inequalities, more research is needed to clarify that oral health instructions are effective in preventing tooth loss.

## Supplementary information

**Additional file 1**. Questionnaire for patients used in this study.

## Data Availability

The datasets used and analyzed during the current study are available from the corresponding author on reasonable request.

## Abbreviations

PPD: Probing pocket depth
BOP: Breeding on probing
OR: Odds ratio
CI: Confidence interval
