# Supplementary material for: A multilevel analysis of the importance of oral health instructions for preventing tooth loss: The 8020 Promotion Foundation Study of Japanese Dental Patients
Source: BMC Oral Health. 2020 Nov 18;20:328. doi: 10.1186/s12903-020-01319-9 (PMC7672973; doi:10.1186/s12903-020-01319-9)
Supplement: Supplementary file 1 — Additional file 1. Questionnaire for patients used in this study. [file 12903_2020_1319_MOESM1_ESM.docx]

**Questionnaire for patients used in this study**

| Age | |
| --- | --- |
|  | [ ] years old |
| Gender | |
|  | 1. Male 2. Female |
| Smoking status | |
|  | 1. Current 2. Past 3. Never |
| Current medical history (diabetes mellitus) | |
|  | 1. Yes 2. No |
| Tooth brushing frequency | |
|  | 1. ≥ 3 times per day 2. 2 times per day 3. 1 time per day  4. Several times per week 5. Don’t brush my teeth |
| Use of secondary oral hygiene products (dental floss or interdental brush) | |
|  | 1. Everyday 2. Sometimes 3. Don’t use |
| Bleeding from gums when brushing teeth | |
|  | 1. Always 2. Sometimes 3. No |
| Eating between meals | |
|  | 1. Everyday 2. Sometimes 3. No |
| Reason for dental visit | |
|  | 1. Treatment 2. Maintenance 3. Both treatment and maintenance |
| Years in school (education level) | |
|  | 1. ≤ 9 years 2. 10-12 years 3. 13-15 years 4. ≥ 16 years |
| Subjective economic status | |
|  | 1. Low 2. Lower middle 3. Middle 4. Upper middle 5. High |
| Are you currently working? | |
|  | 1. Yes 2. No |

**Questionnaire for dental clinics used in this study**

| Age of the director of your dental clinic | |
| --- | --- |
|  | [ ] years old |
| Gender of the director of your dental clinic | |
|  | 1. Male 2. Female |
| How many full-time dental hygienists work in your dental clinic? | |
|  | [ ] |
| In the past month, has your dental clinic provided oral health instructions? | |
|  | 1. Yes 2. No |
| If yes to the previous question.  On average, how much time do you spend giving oral health instructions to each patient in your dental clinic? | |
|  | 1. Less than 5 minutes 2. 5-9 minutes 3. 10-19 minutes  4. 20-29 minutes 5. More than 30 minutes 6. Don’t know |
